# A shared-care model of obesity treatment for 3–10 year old children: Protocol for the HopSCOTCH randomised controlled trial

**DOI:** 10.1186/1471-2431-12-39

**Published:** 2012-03-28

**Authors:** Melissa Wake, Kate Lycett, Matthew A Sabin, Jane Gunn, Kay Gibbons, Cathy Hutton, Zoe McCallum, Elissa York, Michael Stringer, Gary Wittert

**Affiliations:** 1 Royal Children’s Hospital, Parkville, VIC, Australia; 2 Murdoch Childrens Research Institute, Parkville, Australia; 3 Department of Paediatrics, University of Melbourne, Parkville, Australia; 4 Department of General Practice, University of Melbourne, Parkville, Australia; 5 Knowsys, Mt Waverley, Australia; 6 Discipline of Medicine, University of Adelaide, Adelaide, Australia; 7 Centre for Community Child Health, Royal Children’s Hospital, Flemington Road, Parkville, VIC, 3052, Australia

## Abstract

**Background:**

Despite record rates of childhood obesity, effective evidence-based treatments remain elusive. While prolonged tertiary specialist clinical input has some individual impact, these services are only available to very few children. Effective treatments that are easily accessible for all overweight and obese children in the community are urgently required. General practitioners are logical care providers for obese children but high-quality trials indicate that, even with substantial training and support, general practitioner care alone will not suffice to improve body mass index (BMI) trajectories. HopSCOTCH (the Shared Care Obesity Trial in Children) will determine whether a shared-care model, in which paediatric obesity specialists co-manage obesity with general practitioners, can improve adiposity in obese children.

**Design:**

Randomised controlled trial nested within a cross-sectional BMI survey conducted across 22 general practices in Melbourne, Australia.

**Participants:**

Children aged 3–10 years identified as obese by Centers for Disease Control criteria at their family practice, and randomised to either a shared-care intervention or usual care.

**Intervention:**

A single multidisciplinary obesity clinic appointment at Melbourne’s Royal Children’s Hospital, followed by regular appointments with the child’s general practitioner over a 12 month period. To support both specialist and general practice consultations, web-based shared-care software was developed to record assessment, set goals and actions, provide information to caregivers, facilitate communication between the two professional groups, and jointly track progress.

**Outcomes:**

*Primary* - change in BMI z-score. *Secondary -* change in percentage fat and waist circumference; health status, body satisfaction and global self-worth.

**Discussion:**

This will be the first efficacy trial of a general-practitioner based, shared-care model of childhood obesity management. If effective, it could greatly improve access to care for obese children.

**Trial Registration:**

Australian New Zealand Clinical Trials Registry ACTRN12608000055303

## Background

Despite recent indications that the upward trend in childhood obesity is plateauing, its prevalence remains at historically high levels [1]. Although childhood obesity affects around 6% (approximately 200,000) of all Australian children, very few of them receive treatment from their general practitioner or paediatrician [2,3]. Effective evidence-based treatments remain scarce and are generally only available to small proportions of seriously obese children through tertiary care settings. Whilst prevention must ultimately be the main goal, there are already a large number of obese children who urgently require effective treatment if the consequences for their adult health – such as heart disease and diabetes, psychological morbidity, and massive excess health care costs – are to be avoided.

So far, the only healthcare setting that is consistently documented to reproducibly improve the body composition and health of obese children is the specialist obesity clinic, generally involving lifestyle advice, motivation and feedback provided by a multidisciplinary team over a year or more. Mean reductions in body mass index (BMI) z-score sustained to at least 12 months are typically around 0.3 [4]; approximately 85% of children typically achieve at least some overall reduction in BMI z-score although only around 30% achieve the reduction of ≥0.5 [4,5] that equates to definite reductions in fat mass [6] and quantifiable improvements in risk factors for heart disease and diabetes [4,7]. Unfortunately, although intervention appears more successful for younger children, the caseload of specialist obesity clinics is often typically skewed towards adolescents with significant psychological, social and family dysfunction for whom treatment is less effective [4]. Furthermore, such clinics are inaccessible to almost all children. By our estimation the nine obesity clinics in children’s hospitals around Australia could see, at most, around 0.05% of affected Australian children each year, and it seems likely that other countries would have similar situations.

Therefore, as the only universally-accessible healthcare service available throughout childhood, general practice might seem the obvious healthcare setting to support the improvement, achievement and maintenance of healthy weight in children who are already overweight or obese. However, trials of obesity approaches in which treatment is initiated and carried out solely by general practitioners, with or without allied health services, have been extraordinarily disappointing to date both for adults [8] and children [9-12]. A new approach is therefore needed to augment the treatment of childhood obesity in primary care.

Nonetheless, there remain good reasons for optimism when considering general practice as a mode for the successful management of paediatric obesity. Firstly, this is where the majority of overweight/obese children present. In a study of 3000 Victorian primary school children, parents reported that 55% of overweight children had attended a GP once or twice in the preceding six months, and 22% three or more times [13]. In two subsequent randomised controlled trials, we have also shown that: (1) general practitioners can and do take up training to offer a series of structured consultations using strategies for family lifestyle change, (2) that they are able to systematically identify children in the overweight and obese categories, (3) that families are willing for their children to be screened for BMI and not only engage, but persist, with their general practitioner, and (4) that this approach does not appear to be harmful for overweight or mildly obese children [10,14]. GPs are very clear that the management of childhood obesity falls within their role [15] and with training they can feel comfort and competent in this area [16].

The literature on shared-care approaches incorporating primary and specialist partnerships is relatively limited but encouraging in achieving similar [17,18] or better [19] disease outcomes with important ancillary outcomes such as increased satisfaction [20] and reduced waiting times [21]. For instance, an adult rheumatoid arthritis trial demonstrated higher quality-adjusted life-years for the shared-care than the aggressive arm [18], while a shared-care intervention for patients newly diagnosed with cancer increased general practitioner contact and positively influenced patients’ attitudes toward the healthcare system [17]; in neither trial was the disease outcome poorer in the shared-care arm. However, few shared-care trials have focused on children, and none on childhood obesity.

Given the potential benefits of health information technology to general practice [22], the Australian government has prioritised its use and value [23] with the result that, by 2005, 80% had broadband access and nearly 90% used a computer for clinical purposes [24], and is most likely close to 100% as of 2011. Some health information technology features are already nearly universally (eg prescribing) or frequently (eg accessing patient educational material) used, but far fewer general practitioners (<20%) are accessing computerised clinical information or using online decision support during consultations [24]. It is clear that e-health has both promise and limitations [25] and that the potential will not be actualised without carefully designing e-health initiatives into the primary care process. Health information technology could present an excellent mechanism to enhance shared-care models.

The HopSCOTCH (Shared Care Obesity Trial in Children) randomised trial is the first to our knowledge to study the efficacy of a general-practitioner based, shared-care model in reducing obesity in children – a population relatively underserved by evidence-based approaches [26]. The intervention needs to be developed in such a way that it could be widely implemented with consistency and sustainability, but with relatively little training. Underpinning this would be a very practical software platform that would provide standalone guidance and information to GPs while also enhancing primary-specialty care partnerships. The software would also support continuing practice improvement activities and the individual practitioner feedback that has proved useful in many fields [27]. Essentially, we hope to replicate the effectiveness of the specialty obesity clinic in the general practice setting, with attention to feasibility, sustainability and a wider and more systematic availability.

## Aims and hypothesises

The **aim of the HopSCOTCH trial** is to develop, implement and trial an innovative shared-care approach to manage childhood obesity. We will compare outcomes for 3–10 year old obese children randomised to a shared-care model (general practitioners working with paediatric obesity specialist, consisting of a paediatrician and dietician - ‘intervention’ group) with outcomes for those receiving usual patient-driven primary care (‘control’ group).

We **hypothesise** that:

1. Compared to the control group, the intervention children will demonstrate better outcomes at 15 months in terms of lower:

i) Relative BMI, measured as a z-score (primary outcome)

ii) Percentage body fat

iii) Waist circumference

2. Compared to the control group, the intervention children will not show evidence of harm (ie poorer health status, body satisfaction, or global self-worth) at 15 months.

3. The intervention will be acceptable and feasible to (i) parents, (ii) general practitioners, (iii) general practice staff, and (iv) the obesity specialists.

## Methods and Design

### Approval and registration

The project is funded by the National Health and Medical Research Council of Australia (Project Grant 491212). It has been approved by the Royal Children’s Hospital Melbourne Human Ethics Committee (28017) and The University of Melbourne Human Research Ethics Committee (0827435).

### Design

**Figure 1:**
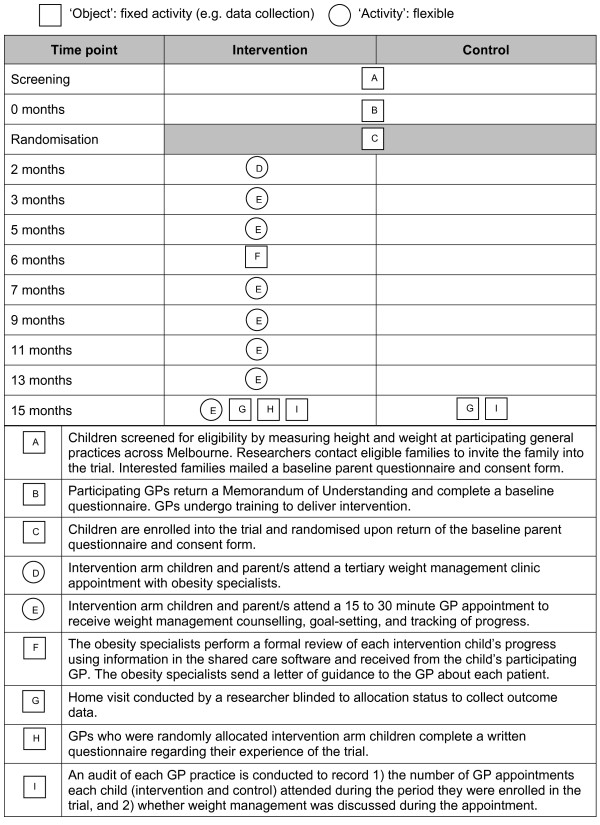
Graphical depiction of components of the HopSCOTCH trial.

HopSCOTCH is a randomised controlled trial of a shared-care intervention versus usual care in obese children (see Figure 1). The trial commenced in April 2008 and will run until December 2011. This period encompasses participant recruitment, baseline data collection, intervention delivery, follow up data collection (15 months post-recruitment, equating to approximately 12 months post-intervention) and data analysis.

### Participants

Participants are (1) 120 children aged 3–10 years, identified as obese according to the United States Centres for Disease Control (CDC) charts (≥95^th^ age- and sex-specific percentile) [28]; and (2) their parents. Children were identified through participating general practitioners (GPs).

### GP recruitment and training

To recruit GPs, HopSCOTCH was widely advertised through the Royal Australian College of General Practitioners and the Victorian general practice research network ‘VicReN’ e-bulletins/newsletters. Personalised invitations were also sent to GPs who previously participated in the LEAP 1 and LEAP 2 trials [10,29]. Of the 70 GPs who initially expressed interest, 35 GPs across 22 practices decided to take part.

GPs attended a 2½ hour group training session for instruction in the “stages of change” model [30] and training in brief, solution-focused family therapy [31]. GPs were shown role model scenarios of GPs using solution-focused therapy in consultations for healthy family lifestyle and given the opportunity to mimic these skills themselves with colleagues in role play scenarios designed by the research team. GPs also completed readings on current obesity management, followed by a brief online quiz to test their knowledge and training in measuring protocols. Those delivering the intervention received one-on-one training in how to use the specially designed shared-care software. GPs were provided with the following remuneration for their time and for bulk-billing all shared-care patients: $220 for attending the training sessions; $25 per child recruited from their practice; and $75 for GPs that saw shared-care patients five times and an additional $75 if they saw them eight or more times.

### Recruitment of children

To recruit children, HopSCOTCH was publicised in participating practices through posters, brochures and practice staff. Trained general practice staff opportunistically offered to weigh and measure children using calibrated digital scales and rigid stadiometers supplied by the research team. Eleven practices also sent letters to all of their in-age children inviting them to attend a dedicated weigh and measure session run by the research team or practice staff at the practice. With parent assent, their contact details and child anthropometry data were then mailed or faxed to the research team. Upon receipt, the research team calculated each child’s BMI and BMI percentile. Provided parents had left contact details, ineligible children’s families were sent a letter informing them of their ineligibility, while eligible families were telephoned to propose participation in the trial. Interested eligible families were mailed a parent information sheet, consent form and parent baseline questionnaire. Upon receipt of the written informed consent and the parent baseline questionnaire, families were enrolled in the trial.

### Inclusion and exclusion criteria

#### *Inclusion criteria*

Eligible families included children who met both the following requirements:

1) BMI ≥95^th^ for age- and sex-specific percentile according to the CDC charts, placing them in the obese range; and

2) aged between 3 and 10 years (i.e. up to but not including their 11^th^ birthday).

#### *Exclusion criteria*

Children were excluded if they met any of the following criteria:

1) receiving ongoing weight management in a secondary or tertiary care program;

2) a known endocrine or genetic cause for their obesity;

3) a major disability or health condition judged by parents and/or researchers to preclude meaningful participation;

4) their family did not speak sufficient English to complete questionnaires and participate in the trial.

#### *Randomisation and blinding*

Randomisation occurred via a concealed, computerised random number sequence stratified by general practitioner and pre-generated by the Clinical Epidemiology and Biostatistics Unit at the Royal Children’s Hospital. Once enrolled (i.e. on receipt of written informed consent and baseline questionnaire) a research assistant, who was not otherwise involved with the trial, randomised children to either the shared-care or usual-care arm. All families were advised of their child’s allocation by a mailed letter. All outcome measures were collected by researchers blinded to the child’s allocation status.

### Intervention arm

#### *Shared-care software*

A web-based shared-care software was designed with the goals of 1) allowing the obesity specialists and GPs to collaborate and communicate closely in the care of their patients, 2) providing a structured yet efficient approach to weight management care, and 3) providing a mechanism that allows both GPs and specialists to record and track patient progress simultaneously. The software’s weight management care plan consists of five steps: 1) recording anthropometry, 2) reviewing BMI change using an online chart to plot and track BMI visually over time against percentile charts, 3) assessing and tracking progress and motivation, 4) reviewing care plan (i.e. issues and goals), and 5) providing educational resources.

The HopSCOTCH software, developed in collaboration with Pen Computer Systems Pty Ltd (PCS), was designed to support specialist and GP management of children with obesity and to facilitate communication of information between the health care providers involved. Specifically, the HopSCOTCH system provides notification and communication between providers of care, access to patient information for care team members via the shared web-based HopSCOTCH record, obesity assessment and management tools, and help in developing a detailed management plan and history. Printouts of plans, educational resources and patient summaries can also be obtained. HopSCOTCH is launched from a desktop application, the PrimaryCare Sidebar®, a proprietary product of PCS. The PrimaryCare Sidebar® sits on the right of the screen (by default) and contains a series of panels, each with links to a range of primary care software tools. The panels allow the tools to be grouped into logical areas of health care. The HopSCOTCH system is accessed via the PCS Linked Care^TM^ panel.

### Obesity specialist consultation

The shared-care intervention involved each family (at a minimum the index child and one parent/guardian) attending a single one-hour session at Melbourne’s Royal Children’s Hospital, where they saw the obesity specialist team comprising a paediatrician and a paediatric dietician who specialise in childhood obesity and weight management. Prior to the appointment, researchers extracted clinically-relevant information about the child and family from the baseline questionnaire, including family history, medical history, daily diet, physical activity and sedentary activities, and scored multi-item scales (see Additional file 1). These data were entered into a summary, with abnormal values flagged, in order to both save time on history-taking and to provide additional information that would not normally be available during a single first clinical consultation (see completed example Additional file 1). This allowed the team to devote more consultation time exploring lifestyle modification, rather than primarily information gathering.

At the appointment, a researcher first measured the child’s height, weight and percentage of truncal fat. The paediatrician then interviewed the child and family, taking a clinical history and examining the child using a standard protocol to identify possible causes and co-morbidities of obesity. The dietician then undertook a detailed dietary history and outlined general principles of healthy eating, offering targeted advice based on the child and family’s eating patterns. Physical and sedentary activities were assessed and, together, the paediatrician and dietician then discussed the lifestyle changes required that would most likely assist in successful weight management for the child. This advice focussed on family change and support, in accordance with research showing beneficial results to the child when the parents are involved [32], and is consistent with current recommendations that, for most obese children, BMI reduction is best achieved by maintaining, rather than losing, weight as the child grows [33]. Details of the specialist consultation, including the clinical summary and pathology results (if applicable) and an initial care plan, were then entered into the shared-care software.

If clinically indicated, parents/guardians were asked to bring their child to the Pathology Department at the Royal Children’s Hospital within the next 2–3 weeks for standard metabolic tests such as a check of thyroid function. These results were also entered into the shared-care software so the GP could access them.

### GP consultations

After the specialist appointment, a follow-up appointment with the child’s GP was scheduled by the research team. Both the specialist and research team encouraged families to see their GP for regular (i.e. every 4–8 weeks) weight management consultations for a year following the specialist consultation. Information from the obesity specialists was available to all GPs via the shared-care software, including the family’s customised care plan.

The weight management GP consultations were designed to: 1) review lifestyle and BMI progress; 2) identify and solve problems where possible; and 3) set new goals using brief solution-focused techniques. The obesity specialists were available to the GP on an ‘as-needed’ basis throughout the trial. At the 6-month point, the obesity specialists formally reviewed each family’s progress using the synchronised software, with a focus on solutions and guidance for GPs. Each family’s review generated a one-page letter that was sent to the treating GP.

### Control arm

Participants in the usual-care (control) arm were not offered an obesity clinic appointment or identified as being in the trial to their GP. Parents were informed that they were free to seek assistance with their GP or with any other service. Should they present to their GP, the GP would be able to implement their usual clinical care and utilise skills gained in the training process, but they were not able to access the shared-care software to track progress, educational resources or access support from the obesity specialists regarding these patients.

### Measures and training

Table 1 summarises all outcome measures for the trial, with the primary outcome being BMI expressed as kg/m^2^[33]. All outcomes will be measured at 15 months post-randomisation, equating to approximately 12 months after the clinical consultation for the intervention children.

**Table 1:** Primary and secondary outcome measures for the HopSCOTCH trial

Construct	**Time Point**		**Measure**	**Additional information**
	**Baseline**	**Outcome**		
**Primary Outcome**				
Body Mass Index (kg/m^2^)	**•**	**•**	Portable rigid stadiometer (model IP0955, Invicta, Leicester, UK); measured Calibrated digital scale (model TITHD646, Tanita, Toyko, Japan); measured	Height is measured twice and the average used; if the values differ by >0.5 cm a third measurement is taken and the average of the two closest values used. Weight, while wearing light clothing, is measured once at baseline, and measured twice at outcome. Average weight used at outcome; if the values differ by ≥0.2 kg a third measure was taken and average of the two closest values used. BMI is calculated as weight (kg)/(height (m)2). BMI z-score is calculated according to the US Centers for Disease Control (CDC) reference values [28], using the Stata ‘zanthro’ function.
**Secondary Outcomes**				
Waist circumference		**•**	Lufkin Executive Steel Tape (W606PM); measured	Average of two waist measurements; if they differ by ≥1 cm, a third measurement is taken and the mean of the two closest used.
Body fat (%)		**•**	Tanita Digital Body Composition Monitor (BC-351)[37]; measured	Average of two body percentage fat measurements.
Blood pressure/ heart rate		**•**	Welch Allyn ProBP3400; measured	Three blood pressure/heart rate readings are taken at least two minutes apart on the right arm with the child sitting; the average of the two closest readings is used.
Nutrition		**•**	4 day food diary; parent report	Parents report child’s consumption of each of 17 food and drink items (0, 1, 2, >2 times) for two weekdays and two weekend days. Dichotomous (“yes” v “no”) variables are derived for five “healthy behaviours” (high fruit, vegetables, and water; low fatty/sugary foods and non-diet sweet drinks) for each day. The number of healthy behaviours per day are summed to give a score between 0 and 5 (higher score indicating more healthy behaviour).
Physical activity		**•**	Actical Accelerometer (Mini Mitter); measured	Worn for 7 full days; ≥5 valid days required. Valid days have ≥10 hours of non-missing data between 6 am-11 pm. Missing data are segments with ≥20 minutes of consecutive “0” counts, or counts >0 that are constant for ≥10 minutes. Outcomes across all valid days: mean activity counts/min, and % time spent in moderate to vigorous physical activity.
Health status	**•**	**•**	Paediatric quality of life inventory (PedsQL 4.0); self report and parent-proxy versions [38]	Parent-completed 23-item scale that yields total, physical summary, and psychosocial summary scores, each with a possible range of 0–100 (100 = best possible health); quantitative variable.
Body dissatisfaction		**•**	Body figure perception questionnaire; self report [39]	Child picture scale of 1–7 (1 = underweight, 7 = obese) from which child picks perceived and ideal selves. “Perceived” minus “Ideal” self yields a discrepancy index, with positive and negatives scores representing desires to be thinner and fatter, respectively.
Physical appearance and self worth		**•**	Modified from Harter’s perceived competence scale; self report	Six pairs of statements with binary response format; children choose the statement from each pair that is closest to their competence. Each of the 6 responses is then coded as being either “positive/better perception” or “negative/worse perception”. The 6 responses are analysed as a single outcome.
Behaviour		**•**	Strengths and difficulties questionnaire [40]; self report	Parent-completed 25-item scale that yields scores for conduct problems, emotional symptoms, hyperactivity, peer relationships and pro-social behaviour.
Parent Readiness to change	**•**	**•**	Parent’s readiness to change child’s weight[41]; self report	3 items, each with a possible 5 responses (strongly agree – strongly disagree).
Parent BMI		**•**	Weight (kg)/(height (m)2); measured and self report	Baseline values reported for self and partner by responding parent. Values at 12 months measured for the parent(s) present with the child and reported; measured data used preferentially.

All researchers involved in baseline and outcome measurements were trained by researchers experienced in conducting similar measurements in the community from other research trials and longitudinal studies in childhood obesity. Researchers were trained at a single one-hour session where each measurement was demonstrated and repeatedly practised to ensure accuracy, competency, and reliability.

### Process evaluation

Process evaluation will be completed by parents and GPs. The items will document extent to which interventions were implemented, acceptability, barriers to attendance, and perceived harms and benefits. Parents will report other assistance received (source, type, intensity) for their children’s weight status.

### Economic evaluation

If the intervention is effective, we will proceed to a full economic evaluation. This will comprise !analysis conducted from both societal and health care perspectives [34], as interventions cost-effective from a health care perspective can add substantially to family costs [35]. This will compare any incremental costs of the intervention (over the control group) to all incremental outcomes detailed above. Resources used in intervention design, development and delivery have been prospectively documented via research team records, the trial database, hospital and general practice records, and parental report and valued using existing unit cost estimates. Uncertainty in the cost and outcome data and sensitivity of results to the evaluation methods chosen will be tested through extensive sensitivity analyses.

### Sample size

The target sample size was calculated to detect a mean difference of 0.3 BMI z-score units at 15 months (comparable to published mean changes seen from specialist obesity clinics[4]) between arms with 80% power at 5% (2 sided) level of significance. Allowing for 10% loss to follow-up, we aimed to recruit 172 children.

### Data Analysis

Analyses will be by ‘intention to treat’ at the level of the individual child. Linear regression will be used to compare quantitative outcomes between the trial groups adjusting for confounders and baseline measures of the outcomes where these are available, using an analysis of covariance approach. Logistic regression will be used to compare dichotomous outcomes.

Confounders selected *a priori* for multivariable models will include child sex, age at randomisation, and family socioeconomic status, which will be assigned according to postal code of residence using the Index of Relative Socioeconomic Disadvantage (mean 1000, s.d. 100) from the Australian Bureau of Statistics census-based Socio-Economic Indexes for Areas (SEIFA) [36].

## Discussion

Without more effective evidence-based treatments to reduce the childhood obesity, we are heading into uncharted territory. Large numbers of obese children are now reaching adulthood, with yet-to-be-quantified impacts on obesity-related comorbidities such as diabetes, poor mental health, hypertension, heart disease and cancers - which would in turn lead to increased health services costs.

If effective, shared-care models for childhood obesity have the potential to offer obese children effective treatment that is easily accessible. Benefits would include increased general practitioner identification of childhood obesity; a shift in focus towards younger obese children (for whom treatment is more effective and secondary prevention of morbidity is still possible); and a model for sustainable, supported partnerships between primary and specialist care with substantially better results than the disappointing stand-alone primary care trials to date.

## Abbreviations

GP: General practitioner; CDC: Centres for Disease Control; BMI: Body mass index; PCS: Pen Computer Systems Pty Ltd.

## Competing interests

All authors declare that they and their spouses, partners or children have no financial and non-financial relationships or interests that may be relevant to the submitted work. The authors declare they have no competing interests.

## Author’s contributions

MW conceived the trial. KL participated in the coordination of the study and drafted the current manuscript, supervised by MW. MAS contributed to the study design, particularly the structure of the specialist obesity clinic. JG contributed to the study design, particularly the general practitioners involvement. KG contributed to the study design, particularly the structure of the specialist obesity clinic. CH contributed to the study design, particularly the general practitioners involvement. ZM contributed to the study design, particularly the structure of the specialist obesity clinic. EY participated in the coordination of the study and drafted the current manuscript, supervised by MW. MS contributed to the study design, particularly the software design and implementation. GW contributed to the study design, particularly the general practitioners involvement and the software development. All authors contributed, read and approved the final manuscript.

## Pre-publication history

The pre-publication history for this paper can be accessed here:

http://www.biomedcentral.com/1471-2431/12/39/prepub

## Supplementary Material

Additional file 1 HopSCOTCH Pre-Specialist Summary. Click here for file
