# Supplementary material for: A shared-care model of obesity treatment for 3–10 year old children: Protocol for the HopSCOTCH randomised controlled trial
Source: BMC Pediatr. 2012 Mar 28;12:39. doi: 10.1186/1471-2431-12-39 (PMC3464143; doi:10.1186/1471-2431-12-39)
Supplement: Additional file 1 — HopSCOTCH Pre-Specialist Summary. [file 1471-2431-12-39-S1.pdf]

HOPSCOTCH Pre-Specialist Summary

Summary Report for Test Patient

Patient Name: Test Patient (Male,10) HOPSCOTCH ID: 90000000

Date Completed:	10 November 2011	Completed by:	Mother
ATSI Status:	No	Main language spoken at home:	English

General Health, Mental Health, and Behaviour

General Health:	Very Good
PedsQL Physical Health:	No Problem
PedsQL Psychosocial Health:	No Problem
SDQ Total Problems:	Normal
SDQ Prosocial Behaviour:	Normal
SDQ Emotional Problems:	Borderline
SDQ Peer Problems:	Normal
SDQ Conduct Problems:	Normal
SDQ Hyperactivity/Inattention:	Normal

Child at School:	Yes
Grade:	3
Repeated a school grade:	Yes
Child at mainstream school:	No
Require teacher's aide:	No

Infant Information

Birth weight:	3200 grams
Birth weight assesment:	Normal BW
Gestation:	39 weeks
Gestation assesment:	Normal Term
Discharged at:	5 days
Type of birth:	Caesarean
NICU/SCN:	No
Ventilated:	

Breastfed for:	9 months
Solids started at:	6 months
Poor weight gain/feeding problems in first two years:	No
Immunisations up to date:	Up to date

Concern about child's weight

Parent:	Very	Help Sought:	No
Child:	A little		

Health Problems

PedsQL impact of child's health on whole family:	No Problem		
Asthma needing medication:	Yes		
Mobility and/or joint problems:	No		
Other hospital admissions:	Yes	Details:	Adenoids removal
Prescribed regular medications:	No		

Allergies:	No		
Developmental delay:	No		
Operations:	No		
Other serious illness:	No		
Steroid medication (current):	No		
Steroid medication (ever):	No		

Child's Family History

	Mother's side	Father's side
High blood pressure:	Mother	Grandmother
Heart attack:		
Diabetes:		
Stroke:		
Lap band/weight reduction surgery:	Grandfather	
Early servere obesity:		
High cholesterol or triglycerides:		Mother; Grandmother
Polycystic ovary syndrome:		
Thyroid problems:		Grandmother

Parent Health Now

General Health:	Excellent
Life difficulty:	Some
Serious psychological distress (K6):	No
Illness/Health conditions:	No
Weight:	72 kg
Height:	164 cm
BMI:	26
BMI status:	Overweight

Parent Health In Pregnancy

Weight gain:	12 kg
Weight gain assessment:	Adequate
Back to pre-pregnant weight:	Yes
Problems during pregnancy:	None
Pregnancy problem details:	

Spouse/Partner

Live with parent and child:	Yes
Relationships to child:	Father
Weight:	99 kg
BMI:	27

Spouse gender:	Male
General health:	Excellent
Height:	189 cm
BMI Status:	Overweight

Child Care

Who else play an important role in looking after child	Grandparents: 5 hours per week.

Child Sleep

Bedtime (weekday):	10 pm
Regular Bedtime:	Sometimes

Amount of Sleep/night	9 hours
Sleep assessment:	Short

Sleep problems >=4 nights a week:	Difficulty getting to sleep at night;<br>Not happy to sleep alone;<br>Waking during the night		Sleep problem:	Moderate

Child's Diet

Regular buy			Days of week (typical week)	
Low fat or reduced fat milk:	Yes		Eat breakfast:	7 days
Softdrink/Cordial/Fruit juice:	Yes		Eat dinner at table with parents:	7 days
Diet Softdrink/Cordial:	Yes		Watch TV while eating meals/snack:	4 days
Single-serve package snack food:	Yes		Eat takeaway meals:	5 days

Child's activity

Days of week during typical week

Watch TV/DVD in own room:	0 days		Distance home to creche/kinder/school:	2 km
Do organised sport or physical activity:	2 days		Safe front or backyard where child can play:	Yes
Attend creche/kinder/school:	5 days		Parent has to be present when child in yard:	No
Walk to/from creche/kinder/school:	5 days		Child has access to other safe play areas:	Yes
Bike/scooter to/from creche/kinder/school:	0 days			
Child use public transportation:	0 days			

Hours per day child spends	On creche/kinder/school days	On non-creche/kinder/school days
Outdoors for transport?	0 hours 25 mins	0 hours 0 mins
Outdoors for play/recreation?	0 hours 30 mins	3 hours 0 mins
Watching TV or DVD?	5 hours 30 mins	6 hours 0 mins
On the computer?	1 hours 30 mins	3 hours 0 mins
Playing video games?	0 hours 0 mins	0 hours 0 mins
